# Comprehensive evaluation of the impact of workplace exposures on physician-certified sick leave in the general working population

**DOI:** 10.1186/s12889-024-17662-3

**Published:** 2024-01-17

**Authors:** Tom Sterud, Andrea R Marti, Eirik Degerud

**Affiliations:** Department of Occupational Health Surveillance, National Institute of Occupational Health, PO Box 8149 Dep, Oslo, N-0033 Norway

**Keywords:** Sickness absence, Work, Occupational health, Occupational exposure, Working conditions

## Abstract

**Background:**

Our objective was to quantify the prospective associations between work factors across chemical, physical, mechanical, and psychosocial domains and the onset of medically certified sick leave.

**Methods:**

Eligible respondents were interviewed in 2009, 2013, or 2016 and were registered in the national sick leave register with an employee relationship lasting more than 50 working days during the year of the survey interviews and the following year (*n* = 15,294 observations). To focus on the onset of high-level sick leave (HLSL; >16 days a year), we excluded individuals with HLSL during the survey year (baseline). We then used mixed-effect logistic regression models to assess prospective associations between self-reported work conditions and the occurrence of doctor-certified HLSL in the following year.

**Results:**

The average occurrence of HLSL was 13.1%. After adjusting for sex, age, level of education, chronic health problems, and smoking, we observed an exposure-response relationship between cumulative exposure to work factors within all domains and the occurrence of HLSL. When evaluating the impact of combined exposures, predicted odds ratios (OR) for employees exposed to 1, 2, and 3 or more work factors within all domains were 1.60 (95%CI 1.32 − 1.94), 2.56 (95%CI 1.73 − 3.74) and 4.09 (95%CI 2.28 − 7.25), compared to those not exposed.

**Conclusions:**

The results support the notion that exposure to multiple work factors in various domains, including psychosocial, mechanical, chemical, and physical work conditions, is associated with an increased risk of high-level sick leave. Employers and occupational health professionals should consider the joint impact of these domains when designing interventions.

**Supplementary Information:**

The online version contains supplementary material available at 10.1186/s12889-024-17662-3.

## Introduction

A high level of sick leave serves as a general indicator of poor health and limited functioning and is closely related to the risk of early departure from the workforce [1, 2]. For society and workplaces, sick leave constitutes a loss of productivity and increased costs. For employees and their families, it can lead to financial and social difficulties. The causes of sick leave are multifaceted and extend beyond medical conditions alone [3]. Working conditions can significantly contribute to the deterioration of health and, in some cases, impose workplace factors that are temporarily or permanently incompatible with an individual’s current health status and ability to perform their tasks [4, 5]. Identifying and addressing these workplace factors that impact sick leave is essential for developing effective interventions to mitigate sick leave, improve productivity, and to provide a healthier and more supportive work environment.

Workplace factors that influence health and sickness absence are commonly categorized into the domains of psychosocial, (bio)mechanical, physical, and chemical work factors [6]. In recent years, an increasing number of prospective studies have substantiated the importance of psychosocial work factors for sick leave [7, 8], including low job control and job strain [8], emotional demands [9–11], role conflict [7, 11, 12] and poor leadership support. Similarly, mechanical work factors, including heavy physical work, heavy lifting, flexion of the back or neck, working with arms above the shoulders, and working primarily standing or squatting [13–17], have been well-documented risk factors for sick leave. In contrast, workplace factors within the domains of physical and chemical working conditions have been less frequently studied in relation to sick leave [16, 18]. Some studies have reported that combined measures of ‘hazardous working conditions’ (dirt and dust, dampness, noise, solvents, or other irritating substances) [19–21], and specific factors, including excessive noise [22, 23], body vibrations [14, 24], and skin exposure to cleaning products [19], are associated with a higher level of sick leave in studies of the general working population.

A noticeable feature of the literature referenced above is that the different work factors have been studied as individual factors often limited to psychosocial or mechanical working conditions, which may not fully capture the complexity of how the working environment influences health and sick leave. In many real-world scenarios, workers are likely exposed to multiple work factors that span the domains of psychosocial, mechanical, chemical, and physical working conditions. Although some studies have investigated the risk of long-term sick leave associated with exposure to multiple mechanical factors [13] or combined psychosocial work factors [25], and joint exposure to psychosocial and mechanical work factors [26, 27], none of these studies have considered work factors within the domains of physical and chemical working conditions. Furthermore, to our knowledge, there are no studies that have evaluated the risk of sick leave associated with exposure to combinations of work factors from different domains of working conditions, which could provide a more comprehensive understanding of the exposure situation and offer a solid foundation for designing preventive measures.

In the current study, our aim is to address certain limitations observed in the existing literature. We use longitudinal data to explore the prospective associations between work factors that encompass chemical, physical, mechanical, and psychosocial working conditions, and the likelihood of high-level medically certified sick leave in the general working population. Building on our previous studies [11, 14, 19], our objective was to gain a more nuanced understanding of how different work factors interact and contribute to the risk of sick leave. To achieve this, we adopted a three-step approach. Initially, we assessed the associations between sick leave and specific work exposures within each of the four domains. Second, we establish four indices to measure cumulative exposure to work factors within each domain (“cumulative exposure”) and examine their association with subsequent sick leave. Lastly, we assessed the effect of exposure to various combinations of work factors across domains (“combined exposure”) on the risk of sick leave.

## Methods

### Study design and population

The Survey of Level of Living-Working Conditions is an ongoing longitudinal survey of a representative sample of Norwegian residents aged 18–66 years, conducted by Statistics Norway every three years, primarily through personal telephone interviews (with only 0.5% of interviews completed face-to-face). For this study, we used data from three consecutive surveys, as described in Table 1. The first survey (data collection: June - January 2009/10) involved 12,255 interviews out of a gross sample of 20,136 (60.9%) randomly drawn from the population. In the second survey (April - January 2013/14), the same gross sample was invited to participate, and 10,875 individuals responded (53.1%). For the third survey (September - April 2016/17), two-thirds of the original gross sample was re-invited, and the remaining one-third was replaced with a new random subsample due to a planned rotation of the panel selection. In total, 10,655 interviews (52.6%) were conducted. To ensure the representativeness of the sample, participants aged 17, 18, and 19 years, as well as immigrants, were supplemented at each iteration of the survey. Thus, an individual can participate in one, two or all three surveys (see Table 1). Statistics Norway reported minimal differences between respondents and the gross sample in terms of age, sex, and region, indicating a high degree of representativeness [28].

**Table 1:** Description of the sample

	Sample per survey			Sample in total	
	2009	2013	2016	Observations	Individuals
Gross sample ^a^	20 136	20 492	20 272	60 900*	
Net sample ^b^	12 255	10 875	10 665	33 795	20,341
Response percentage ^c^	60.9%	53.1%	52.6%	55.5%	
Working population sample ^d^	9279	8375	8329	25,983	15,866^d^
Active employee relationship of at least 50 days ^e^	7709	7077	7302	22,088	13,731
Eligible sample ^f^				21,852	13,473
Final sample ^g^				15 294	10,553

a = random-drawn population sample (* maximum number of possible observations)

b = total number of respondents including employed and non-employed individuals

c = respondents who were in paid work for at least one hour during the interview week or were temporarily absent from such work were interviewed about working conditions

d = sum of individuals that were interviewed about working conditions in one survey (*n* = 8504), two surveys (*n* = 4607) and three surveys (*n* = 2755)

e = registered with an active employee relationship of at least 50 actual working days in the survey year and the following year in the sickness absence register

f = eligible sample after deletion of respondents with missing values (*n* = 258 (1.9%) individuals)

g = we used the baseline to remove individuals with a high level of sick leave during the survey year (that is, the baseline year), and we also omitted people with a low level of sick leave (LLSL; 1–16 days each year) at follow-up

As shown in Table 1, from the three consecutive surveys, we selected respondents who reported being in paid work for a minimum of one hour or reported temporary work absence during the interview week (i.e., working population). Additionally, respondents were required to have a documented employee relationship spanning at least 50 working days in both the survey year and the following year, as confirmed by the Norwegian Labour and Welfare Administration’s sickness benefit register. The current study employs a prospective design with a one-year follow-up period for each observation in this register. In the final sample, to facilitate the study of HLSL onset, we utilized baseline data to exclude individuals who already had HLSL during the survey year. This step was taken to further mitigate selection bias, as individuals more susceptible to sick leave might be more likely to be chosen for jobs with higher levels of exposure. We excluded respondents who were self-employed without employees and those with missing values in the exposure variables and covariates. Additionally, we excluded individuals with a low level of sick leave (LLSL), which ranged from 1 to 16 days per year, during follow-up, to specifically concentrate on identifying risk factors for HLSL.

### Measurements

#### Outcome

Sick leave was recorded as the count of doctor-certified sick leave days within each calendar year in the Norwegian Labour and Welfare Administration’s sickness benefit register (specifically, 2010, 2014, and 2017, which constituted the follow-up period for each of the surveys). Due to skewness and clustering around zero, the variable was recoded into a categorical variable. Our focus in this study was high-level sick leave (HLSL), which aligns with the predominant literature that examines long-term sick leave (7, 13, 22, 25, 28). The rationale behind our emphasis on HLSL stems from the recognition that short-term sick leave can often be attributed to minor illnesses or temporary discomforts, which might not necessarily be related to work conditions. In contrast, subpar working conditions tend to accumulate and exert chronic effects on an individual’s health over time, potentially leading to prolonged sick leave, a marker often associated with more severe health issues.

In this study, we defined HLSL as sick leave extending beyond 16 days in the calendar year following each survey. Notably, there is no universally accepted definition of a high level of sick leave (17). Our choice of a 16-day threshold was guided by two reasons: First, it allowed us to distinguish between sick absence days paid by the employer (16 days or less) and sickness absence days paid by the Norwegian Labour and Welfare Administration (more than 16 days). Second, the selected threshold closely approximated the median number of sick leave days among respondents who reported sick leave (specifically, 15 days).

#### Exposure

We obtained self-reported data on exposure to twenty-two individual work factors through a questionnaire. These work factors were grouped into four domains. Psychosocial work factors (comprising five factors): low job control, job strain (resulting from low control and high job demands), role conflicts, emotional demands, and supportive leadership. Mechanical factors (six factors): hand/arm repetition, squatting/kneeling, standing, working with the upper body bent forward, and awkward lifting. Physical factors (five factors): whole-body vibration, hand/arm vibration, noise, heat, and cold. Chemical factors (six factors): contact with oil or lubricants, skin contact with cleaning agents/disinfectants, wet work, exposure to dust or smoke, metals/minerals/organic dust, and gases/vapours.

All measures were dichotomised into “low exposure” or “high exposure” (range 0 − 1). Psychosocial factors were measured using 5-point Likert scales and recoded as low/medium vs. high exposure, where a score > 3 indicated high exposure. Mechanical, physical and chemical work factors were assessed using initial single-item questions with “yes” or “no” responses. Those who responded with “yes” were also asked to estimate the proportion of their working day in which they were exposed to these factors. Different cut-off points were used for various variables, depending on whether a short or long duration of daily exposure was deemed most relevant (for detailed question formulations, answer categories, and cut-offs, see the Supplementary Table). To capture a comprehensive measure of cumulative exposure within each domain of working conditions, we calculated a categorical index ranging from no exposure to exposure to 1, 2, 3, or more factors for psychosocial, mechanical, physical and chemical working conditions (range 0 − 3). Additionally, for descriptive purposes (as shown in Table 1), we devised a measure of exposure to at least one work factor within each domain of working conditions.

#### Covariates

Sex, age, level of education, number of actual working days, and baseline sick leave were based on administrative registry data. Age and education level (based on The Norwegian Standard Classification of Education (NUS)) were treated as continuous variables in the regression analyses but recoded as dummy variables for descriptive purposes (i.e., NUS 0 − 2 Elementary level; NUS 3 − 5: upper secondary education; NUS 6: University/college 4 y; and NUS 7 − 8: University/college 4 y +).

We assessed chronic health conditions using two items: “Do you have any long-term illnesses or health problems? This includes any illnesses or problems that may be seasonal or intermittent, with the requirement that the condition must have persisted or be expected to persist for at least 6 months.” “Do you have a disability or health problems resulting from an injury?” Responses to these questions were dichotomously coded into a single variable to indicate the presence or absence of a chronic health condition. Smoking status was determined using the following two questions: “Do you sometimes smoke?” If respondents answered “Yes,” they were further asked “Do you smoke every day or occasionally?” These responses were then recoded to distinguish between regular smokers and non- or occasional smokers (Table 2).

**Table 2:** Prevalence of working conditions (i.e., exposed to at least one work factor within either the domain of psychosocial, mechanical, physical, and chemical conditions) and occurrence of HLSL distributed by covariates

Covariates	N			Psychosocial	Mechanical	Chemical	Physical	HLSL
			Prevalence (> 0) ^§^	Prevalence (> 0) ^§^	Prevalence (> 0) ^§^	Prevalence (> 0) ^§^	(%)	
Total	15,294		40.0	52.1	33.2	23.3	13.1	
Gender								
Men	8639		31.9	47.7	32.2	30.1	9.4	
Women	6655		50.4	57.9	34.8	14.3	17.9	
Age groups								
17–34	3988		45.5	63.5	43.5	30.9	12.8	
35–49	5945		39.4	49.0	30.3	21.3	12.9	
50–66	5361		36.5	47.1	29.1	19.6	13.4	
Education level								
Elementary level	2007		43.0	69.3	51.4	41.0	17.7	
Upper secondary. not finished	1069		35.1	59.4	39.0	27.1	17.6	
Upper secondary education	4799		38.4	58.6	43.9	35.9	13.2	
University/college 4 years	5148		44.5	45.5	23.8	11.9	12.5	
University/college 4 years+	2271		32.2	35.4	14.1	0.05	7.9	
Chronic health condition								
No	11,736		38.3	50.5	31.3	21.9	10.3	
Yes	3558		45.6	57.6	39.9	27.3	21.8	
Smoking								
No	12,065		39.3	50.6	30.9	21.3	11.9	
Sometimes	1411		43.3	53.6	37.5	24.8	13.8	
Regularly	1818		41.6	61.3	45.5	34.1	19.5	
			10.6 (2)*	74.0 (2)*	162.6 (2)*	146.7 (2)*	80.9 (2)*	

N = number of observations

HLSL = high level of sick leave;

§ >0 = exposed to one or more factors within the specified domain of working conditions

*p ≤ 0.05

### Statistical analyses

To examine the prospective correlation between work factors and the subsequent odds of sick leave, we employed generalized linear mixed models (GLMM). Specifically, mixed-effects logistic regression was chosen as our preferred method since it is suitable for analysing non-normally distributed outcome variables clustered within units, as in our case of repeated observations from the same individuals. Additionally, the follow-up time was considered constant for all individuals (one year), given the absence of precise start and stop dates for sick leave periods. Prospective associations were expressed as odds ratios (OR) with 95% confidence intervals (CI). Model #1 was adjusted for sex, age, and the number of actual working days. In Model 2, we also account for educational level, chronic health problems, and smoking. In both models, random intercepts were included to account for the non-independence of measurements within individuals. A significance level of 0.05 was set for our analyses. We performed all statistical computations using R version 3.6.1.

**Fig. 1:**
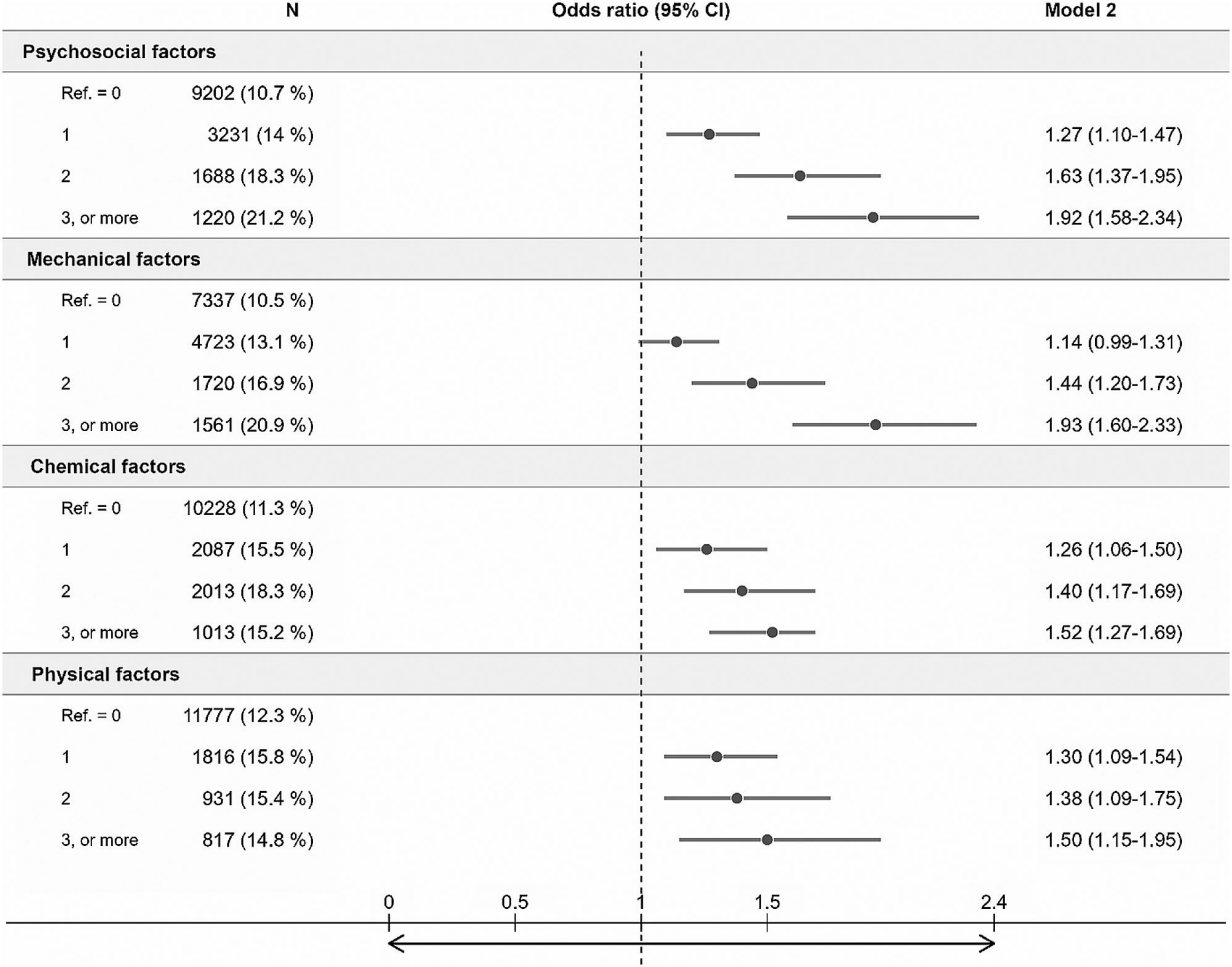
Prevalence and odds ratio of sick leave (HLSL) according to cumulative exposure to psychosocial, mechanical, chemical, and physical work factors

We adopted a three-step approach to investigate the relationship between work factors and HLSL. First, we estimate the OR for each of the 22 specific work factors in relation to HLSL, as detailed in Table 3. Second, we evaluated the OR associated with cumulative exposure to work factors within each domain. This is presented in Fig. 1, where ORs were estimated for categorical variables and continuous variables (reported in the text). Third, we explored the association between «combined exposure» to multiple work factors from different domains of working conditions and HLSL. In this analysis, we combined the chemical and physical domains into a single index, to avoid over-adjustment (i.e., high intercorrelation between physical and chemical factors) and to reduce the number of combinations and interaction terms. We examined two models: the additive model, where the indices were mutually adjusted without interaction terms, and the interaction model, which included interaction terms for all combinations of exposures. These analyses were based on fully adjusted Model #2, as detailed in Table 4. Finally, we used the estimates from the final model to calculate predicted ORs (95% CI), for respondents scoring 0, 1, 2, or 3 on all three indices.

**Table 3:** Individual work factors and their association with High-level sick leave (HLSL) at follow-up (*n* = 15,294)

			Model #1		Model #2	
Work factors	N-exposed (%-exposed)	cases (%) exposed/ the non-exposed)	OR	95%CI	OR	95%CI
**Psychosocial working conditions**						
Low job control	3109 (20.3)	17.9 /11.8	1.55	1.34–1.79	1.44	1.25–1.65
Jobstrain	2128 (13.9)	19.9/11.9	1.76	1.50–2.06	1.64	1.40–1.91
High emotional demands	2775 (18.1)	18.5/11.8	1.60	1.38–1.86	1.54	1.34–1.78
High role conflict	1348 (8.8)	17.8/12.6	1.57	1.30–1.91	1.51	1.25–1.82
Low suportive leadership	1242 (8.1)	17.2/12.7	1.52	1.24–1.86	1.38	1.14–1.68
**Mechanical factors**						
Neck flexion	2533 (16.6)	16.2/12.3	1.46	1.26–1.70	1.25	1.08–1.45
Upper body forward bend	1384 (9.0)	19.72/12.4	1.95	1.61–2.35	1.61	1.34–1.93
Hand-/arm repetition	3198 (20.9)	13.7/12.9	1.05	0.90–1.21	0.96	0.84–1.11
Squatting/kneeling	2033 (13.3)	18.7/12.2	1.95	1.66–2.30	1.60	1.36–1.88
Awkward lifting	1446 (9.5)	22.7/12.1	2.53	2.11–3.03	2.03	1.71–2.42
Standing	3240 (21.2)	17.9/11.8	1.75	1.52–2.02	1.45	1.26–1.67
**Physical factors**						
Whole body vibration	986 (6.4)	13.7/13.0	1.62	1.26–2.08	1.26	0.99–1.61
Hand/arm vibration	1393 (9.1)	14.60/12.9	1.83	1.48–2.27	1.35	1.10–1.67
Noise	1913 (12.5)	14.6/12.8	1.50	1.25–1.79	1.20	1.01–1.43
Heat	600 (3.9)	19.3/12.8	2.07	1.57–2.73	1.58	1.21–2.07
Cold	1594 (10.4)	16.2/12.7	1.74	1.44–2.11	1.34	1.12–1.62
**Chemical factors**						
Skin contact. oil or lubricants	1522 (9.9)	12.8/13.1	1.47	1.19–1.82	1.12	0.91–1.38
Skin contact. cleaning agents/ disinfectants	3159 (20.6)	17.1/12.0	1.61	1.39–1.85	1.33	1.15–1.52
Wet work	2999 (19.6)	18.3/11.7	1.66	1.44–1.92	1.32	1.15–1.52
Dust or smoke. metals	509 (3.3)	13.7/13.0	1.62	1.16–2.26	1.23	0.89–1.69
Gases, fumes	494 (3.2)	16.0/12.9	1.70	1.23–2.35	1.39	1.02–1.88
Mineral or organic dust	898 (5.9)	12.0/13.1	1.31	0.92–1.87	1.10	0.87–1.40

Model#1 adjustment for sex, age and number of actual working days

Model#2 = + education level (continuous), chronic health problems and smoking

Note Model#2 = adjustment for sex, age, and number of actual working days, education level (continuous), chronic health problems, and smoking

**Table 4:** High-level sick leave (HLSL) regressed on the indices for cumulative exposure to psychosocial-, mechanical, chemical/physical work factors

	Additive model§		Interaction model§	
***Work factors (indices)***	*OR*	*95%CI*	*OR*	*95%CI*
1. Psychosocial factors (continuous)	1.22	1.15–1.29	1.26	1.14–1.40
2. Mechanical factors, (continuous)	1.17	1.10–1.25	1.20	1.07–1.34
3. Chemical/physical factors, (continuous)	1.12	1.04–1.20	1.05	0.92–1.20
**Interaction terms**				
Psychosocial *Mechanical			0.93	0.86–1.01
Psychosocial * Chemical/physical			0.98	0.89–1.08
Mechanical* Chemical/physical			1.00	0.93–1.08
Psychosocial * Mechanical* Chemical/physical			1.03	0.98–1.09

§ adjustment for sex, age, and number of actual working days, education level (continuous), chronic health problems and smoking + random intercept. All work factors (indices) are mutually adjusted

## Results

The statistical analyses included a total of 15,294 observations and 10,553 respondents (See Table 1 for sample description). Table 2 presents the prevalence of exposure to at least one work factor within each domain of working conditions, along with the prevalence of sick leave categorized by sex, age, education, chronic health conditions, and smoking status. The results revealed that women were more frequently exposed to one or more factors within the psychosocial and mechanical domains, while men showed a higher prevalence of exposure to one or more factors within the physical domain. The prevalence of chemical work factors was rather similar. Furthermore, younger workers, those with lower levels of education, individuals with chronic health conditions, and smokers tended to be more frequently exposed to one or more factors in all four domains. Regarding HLSL, its overall prevalence was found to be 13.1% (corresponding to 1,999 observations and 1,885 individuals). HLSL was more prevalent among women and individuals with lower educational attainment, chronic health conditions, and smokers.

To assess the associations between sick leave and specific work exposures within each of the four domains, Table 3 presents the prevalence of each work factor within the psychosocial, mechanical, chemical, and physical domains, together with their estimated association with HLSL. The prevalence of work factors within each domain varied, with percentages ranging from 8.1 to 20.3% for psychosocial factors, 9.0–20.9% for mechanical factors, 6.4–12.5% for physical factors, and 2.9–20.6% for chemical factors. In the statistical analyses, we initially adjusted for age, sex, and actual working days (model 1), which revealed that 21 of 22 work factors were significantly associated with the OR of HLSL. Further adjustment for education level, chronic health problems and smoking (model 2) resulted in some attenuation of the OR estimates for factors within the psychosocial and mechanical domain, with a greater attenuation for factors within the physical and chemical domains. In model 2, we observed that 5 of 5 psychosocial factors (with OR ranging from 1.38 to 1.64), 5 of 6 mechanical factors (with OR ranging from 1.21 to 2.07), 4 out of 5 physical factors (with OR ranging from 1.20 to 1.58) and 3 out of 6 chemical factors (with OR ranging from 1.32 to 1.39) were associated with a statistically significant increase in the OR of HLSL (for complete overview of all OR and CI, see Table 3).

To assess cumulative exposure to work factors within each domain Fig. 1 shows ORs (95% CI) depicting the strength of the association between cumulative exposure to work factors within each domain and HLSL. This assessment involved the comparison of groups exposed to 1, 2, or 3 risk factors within a specific domain with the unexposed group in the same domain. The results consistently reveal higher ORs for groups exposed to an increasing number of risk factors, even after adjusting for education level, chronic health problems, and smoking in Model 2, which had diverse effects on estimates in the different domains. Next, we included the indices as continuous variables to test for linear trends and we observed incremental increases in the ORs (95% CI) for each additional risk factor in the four domains: psychosocial factors (OR = 1.27, 95% CI 1.20–1.35), mechanical factors (OR = 1.24, 95% CI 1.17–1.32), chemical factors (OR = 1.17, 95% CI 1.11–1.24) and physical factors (OR = 1.16, 95% CI 1.08–1.25) (table not shown).

To assess the effect of exposure to various combinations of work factors from different domains, Table 4 presents ORs with 95% CI for the risk of HLSL based on a model that consolidates the physical and chemical exposure indices into a single index, resulting in three indices in total.

**Fig. 2:**
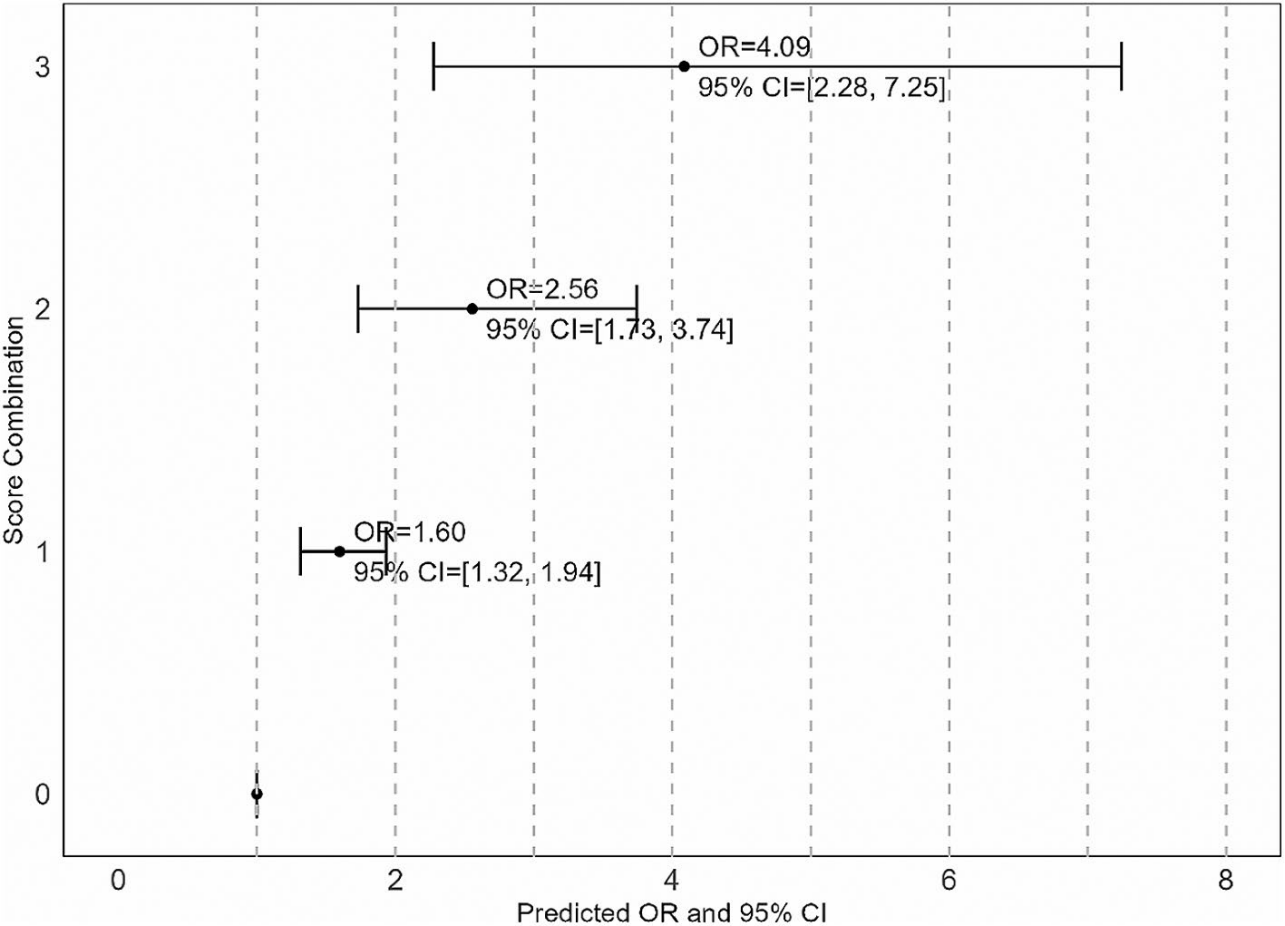
Predicted OR and 95%CI for HLSL based on the additive model in Table 4. *Note:* The reference group consists of individuals unexposed (score = 0) in all three indices, i.e., psychosocial, mechanical, and chemical/physical factors. The estimated odds ratios (ORs) with 95% confidence intervals (CIs) are calculated for respondents with scores of 1, 2, and 3 in each domain. These estimates are adjusted for sex, age, number of actual working days, education level (continuous), chronic health problems, and smoking. Additionally, the three indices are mutually adjusted.

In the ‘additive model,’ all three indices demonstrated independent and statistically significant associations with HLSL. In the ‘interaction model’, most of the interaction terms were near unity and did not reach statistical significance. Based on these findings, we used estimates from the additive model to calculate the OR estimates for combined exposure to work factors across domains. We manually calculated odds ratios (OR) with 95% confidence intervals for respondents who scored 0, 1, 2, or 3 on all three indices. The reference group consisted of unexposed individuals (score = 0) in the three indices. The estimated ORs (95% CI) for the respondents with scores of 1, 2, and 3 in each dimension are shown in Fig. 2.

## Discussion

In this prospective study, we investigated the association between working conditions and register-based sick leave in a representative sample of the general working population. Our findings revealed a significant trend: the risk of doctor-certified sick leave progressively increased with cumulative exposure to work factors in the psychosocial, mechanical, chemical, and physical domains.

Collectively, our results emphasise the importance of focusing on combinations of work factors from various domains when considering risk factors for sick leave in the workplace.

In our initial analysis that focused on specific work exposures within the four domains, we consistently identified associations between five psychosocial factors and five of the six mechanical factors with the onset of HLSL. We observed rather similar ORs for specific work factors within the domain of psychosocial factors. For mechanical factors, the highest OR was observed for lifting in awkward position, while no increased OR was observed for hand / arm repetition. These findings are consistent with previous research and underscore the importance of various work factors for the risk of sick leave. Notable psychosocial risk factors include low job control, job strain, emotional demands, role conflict, and poor leadership support [7–12, 15, 29]. Similarly, mechanical factors such as lifting in strainful positions, neck flexion, and working primarily standing or squatting [13–17] were associated with the risk of sick leave.

Importantly, we also addressed the lesser studied area of physical and chemical work factors in relation to sick leave. Our results contribute to the limited literature on the subject, revealing a higher risk of sick leave associated with exposure to noise, vibrations, and skin contact with cleaning products - in agreement with previous studies that have reported associations between sick leave and noise [22, 23], vibrations [14, 24], and cleaning products [19]. Additionally, we also observed associations between exposure to heat or cold and exposure to gases/fumes with an increased risk of HLSL. These findings underscore the importance of including physical and chemical work factors when assessing the risk of sick leave. It should be noted that the associations between physical or chemical factors and HLSL were somewhat reduced after adjusting for variables such as educational level, chronic health problems, and smoking. Despite this adjustment, our analysis still yielded statistically significant associations. Specifically, even in the most comprehensive model, we identified significant associations between four of the five physical factors and three of the six chemical factors with the risk of HLSL.

As a novel finding, our study reveals that within each of the four domains, the risk of HLSL increases incrementally in a linear dose-response manner for each additional risk factor to which the respondents are exposed. We identified only one previous study in the Danish working population that reported associations between cumulative mechanical exposure and sick leave [13]. Additionally, although previous research has indicated possible connections between combinations of psychosocial factors and concurrent exposure to psychosocial and mechanical work factors, leading to an increased risk of long-term sick leave [25–27], we did not find studies that include consideration of physical and chemical work factors. Furthermore, in accordance with the previous study of cumulative mechanical exposure (15) and combined psychosocial exposure (26), we treated all individual work factors as equally important when we combined them. Although theoretically some of the specific factors included in the cumulative index could be considered more important than others, the present data did not show a clear pattern indicating one or two ‘important’ factors for the risk of HLSL. We observed rather similar ORs for specific work factors within the different domains.

To further explore the impact of combined exposure to work factors from all four domains, we addressed the additive and multiplicative risks associated with exposure to combinations of work factors from psychosocial, mechanical, and physical/chemical domains. Our results support an additive effect, demonstrating that the risk of sick leave increases among employees with combined exposure to multiple work factors from different domains, compared to those exposed to work factors within a single domain. For example, employees exposed to three or more factors of psychosocial, mechanical, physical, or chemical work were estimated to have a 3.8-fold increase in the risk of high-level sick leave compared to the unexposed group. In comparison, employees exposed to three or more psychosocial or mechanical working conditions without other types of work exposure had a 1.9-fold increased risk of high-level sick leave. These findings underscore the importance of considering the cumulative effects of multiple work factors across various domains and their potential impact on employees’ sick leave patterns.

Our findings emphasise the significant impact of various workplace factors on increased sick leave. Recent reports from the European Agency for Safety and Health at Work indicate that due to the shift in the job market towards the service industry, the relative importance of psychosocial stressors is expected to gain prominence due to an increase in social interaction with patients, customers, clients, or colleagues. However, traditional ergonomic risks persist despite changes in work tasks and sectors over the past 10–25 years. These risks involve repetitive movements in industry and service roles, heavy lifting in craft occupations, and uncomfortable positions, which affect a substantial portion of the workforce. Exposure to physical risks such as noise, vibrations, and chemical agents has remained largely unchanged over the past 15 years [30]. To promote healthier and more productive work environments, employers and policymakers must adopt a comprehensive approach to address these factors. Regular monitoring of work conditions and their impact on employee health is essential. Instead of considering psychosocial or physical factors in isolation, our study suggests that a more effective approach for employers and health professionals is to consider the interplay of psychosocial, mechanical, and physical/chemical factors when developing strategies to prevent sick leave.

## Limitations and Strength

This study possesses several strengths that enhance its validity and reliability. These include a large nationwide random sample, a prospective design, and the use of different measurement types for exposure (self-report) and outcome (registry-based sick leave). Non-response examinations conducted by Statistics Norway indicated only minor differences between non-responders, and responders although non-response rates were slightly higher among respondents with an elementary education level [28]. Furthermore, the linkage of survey data with registered sickness absence data minimised participant loss, and previous findings suggested that the small number of participants lost to follow-up is unlikely to substantially affect the results [31].

One possible limitation is the measurement of sick leave as the cumulative number of days during a calendar year, as precise start and stop dates for individual sick leave periods were not available. Although there is a theoretical possibility that several short-term sick leave periods might add up to our definition of high-level sick leave (HLSL), it is important to note that in Norway, employees receive full compensation from the first day of sick leave. Self-certification is allowed for up to three sick leave periods, with some extending up to eight consecutive days. When a single absence period exceeds the specified number of days, a doctor’s certificate is required. Therefore, it is unlikely that many employees with HLSL would have several short-term spells.

Another limitation lies in the self-reporting of exposure data, which opens the possibility of unmeasured factors that influence exposure and sick leave and potentially inflate the estimates. Additionally, studying causal associations between work exposure and sickness absence faces challenges due to different selection processes that may impact observed associations. Two opposing processes may occur: individuals with robust health might accept jobs with difficult working conditions (compensated by higher wages), or healthier individuals might tolerate less favourable conditions and are less likely to leave a risky job (healthy worker effect), leading to underestimated associations. In contrast, resourceful individuals can acquire the best and least risky jobs, while those with poorer health and work ability can end up in jobs associated with a higher sickness absence, leading to overestimated associations. To address these concerns, we took measures to mitigate overestimation, including excluding individuals with HLSL in the baseline year and adjusting for self-reported ‘chronic health problems’ at baseline. Additionally, this approach is likely to reduce reporting bias, as people with poorer health may assess work exposure differently than healthy individuals. Lastly, we adjusted for smoking as a proxy for lifestyle factors that are known to affect the level of sick leave [32]. Lifestyle factors are likely to exhibit an uneven distribution in various job categories and, consequently, may confound the association between working conditions and sick leave. Data on other pertinent lifestyle factors, such as alcohol consumption, body mass index, and physical activity [32], were not available in the data.

## Conclusion

Our study provides new insights into the interplay between work conditions and sick leave. The results support the notion that exposure to multiple work factors in various domains, including psychosocial, mechanical, chemical, and physical work conditions, is associated with an increased risk of high-level sick leave in a dose-response manner. In particular, the highest risk of sick leave was observed among employees exposed to a combination of psychosocial, mechanical, physical, or chemical factors. These findings underscore the importance of implementing comprehensive workplace interventions that address multiple risk factors simultaneously. Recognising the effect of various work factors on sick leave, organisations can develop targeted strategies to improve working conditions and possibly reduce sick leave rates.

### Electronic supplementary material

Below is the link to the electronic supplementary material.

Supplementary Material 1: The Supplementary Table presents detailed formulations of the questions for each of the measures of work factors in the domains of psychosocial, mechanical, physical, and chemical working conditions. In addition to answer categories and cutoff values for the different work factors

## Data Availability

The data that support the findings of this study are available from the Norwegian Centre for Research Data (https://nsd.no/) or Statistics Norway (mikrodata@ssb.no), but restrictions apply to the availability of these data, which were used under license for the current study, and so are not publicly available. Data are however available from the authors upon reasonable request and with permission of the Norwegian Agency for Shared Services in Education and Research (SIKT) and Statistics Norway (SSB).
