# Supplementary material for: Comprehensive evaluation of the impact of workplace exposures on physician-certified sick leave in the general working population
Source: BMC Public Health. 2024 Jan 17;24:214. doi: 10.1186/s12889-024-17662-3 (PMC10795203; doi:10.1186/s12889-024-17662-3)
Supplement: Supplementary file 1 — Supplementary Material 1: The Supplementary Table presents detailed formulations of the questions for each of the measures of work factors in the domains of psychosocial, mechanical, physical, and chemical working conditions. In addition to answer categories and cutoff values for the different work factors [file 12889_2024_17662_MOESM1_ESM.docx]

Supplementary Table 1

| Exposure variabel | questions | Cut off values |
| --- | --- | --- |
|  |  |  |
| **Psychosocial factors** |  |  |
| Low job control | To what extent can you 1) decide work pace?  2) decide when to take breaks?  3) decide how to do your job?  4) influence decisions that are important to your work? | The five-point scales mean  scores were dichotomised into  low / medium (1.0−3.0)  vs  high (3.1−5.0). |
| Jobstrain (high job demands and low job control) | Job strain was defined as the combination of high job demands and low job control, using the cut-off values described in the adjacent column. Job demands were assessed with two items: How often is it necessary to work at a rapid pace? and How often do you have too much to do? Low job control was measured using four items, as described above. |  |
| High role conflict | 1) How often do you receive contradictory requests from two or more different people?  2)How often are tasks given without being given sufficient tools and resources to complete?  3) How often do you have to do things that you think should be done differently? |  |
| Low supportive leadership | 1)“If needed, how often can you get support and help from your immediate superior with your work?  2)“Are your work achievements appreciated by your immediate superior?”  3) Does your immediate superior treat employees fairly and equally? |  |
| High emotional demands§ | 1) To what extent do you need to deal with strong feelings such as sorrow, anger, desperation, frustration, and so on from customers, clients, or other people who are not employed at your workplace?  2) To what extent do you need to conceal negative feelings such as anger, irritation, frustration, etc. for customers, clients, or other people who are not employed at your workplace? |  |
| **Mechanical factors** |  |  |
| Neck flexion | Do you work with your head bent forward? §§ | Yes, 1/4 of the time or more. |
| Upper body forward bend | Do you work in positions where you lean forward without supporting yourself on your hands or arms? §§ | Yes, 1/4 of the time or more. |
| Hand / arm repetition | Does your work involve repeated hand or arm movements? §§ | Yes, 3/4 of the time or more. |
| Squatting / Kneeling | Do you need to squat or kneel during your work? §§ | Yes, 1/4 of the time or more |
| Awkward lifting | Do you have to lift things in uncomfortable positions? §§ | Yes, 1/4 of the time or more. |
| Standinga | Do you work standing up? §§ | Yes, 3/4 of the time or more. |
| **Physical factors** |  |  |
| Whole-body vibration | Are you, in your day-to-day work, exposed to vibrations that cause your whole body to shake, e.g., from a tractor, forklift truck, or other piece of machinery? §§ | Yes, exposed daily |
| hand/arm vibration | Are you, in your day-to-day work, exposed to ... vibrations from machines or  Tools you hold with your hands? | Yes, exposed daily. |
| Noise | Are you, at your day-to-day work, exposed to loud noise so loud that you must stand next to each other?  and shout to be heard? | Yes, exposed daily. |
| Heath | Are you, in your day-to-day work, exposed to heat that is approximately 28 degrees Celsius or more? | Yes, 1/4 of the time or more. |
| Cold | Are you, in your day-to-day work, exposed to cold, i.e. outdoor work in the  wintertime, or working in cold rooms, etc. | Yes, 1/4 of the time or more. |
| **Chemical factors** |  |  |
| skin contact, oil, or lubricants | Are you exposed to skin contact with oils, lubricants, or cutting fluids in your day-to-day work? | Yes, exposed daily. |
| skin contact, cleaning agents/ disinfectants | Are you exposed in your day-to-day work to cleaning products, disinfectants, solvents, or other degreasing agents? | Yes, exposed daily. |
| Wet work | Do you get water on your skin several times a hour at work? Including washing your hand. | Yes, exposed daily |
| Dust or smoke, metals | In your work environment, can you clearly see in the air or smell dust or fumes from metals? eg welding fumes, or lead, chrome, nickel, zinc, aluminium, copper, or tin dust”. | Yes, exposed daily. |
| Mineral dust | In your work environment, can you clearly see in the air, or smell 1) …mineral dust? e.g. stone, quartz, cement, asbestos, or mineral wool | Yes, exposed daily. |
| Organic dust | In your work environment, can you clearly see in the air or smell organic dust? e.g., from textiles, wood, flour, clothes, or animals; and 3) … | Yes, exposed daily |
| Gases/vapours | In your work environment, can you clearly see in the air or smell... gas/steam? For example, ammonia, hydrochloric acid, chlorine, nitrous oxides, formaldehyde, hot water vapour, or sulphur gases? | Yes, exposed daily |
|  |  |  |

§ Answer categories were “very seldom or never,” “rather seldom,” “sometimes,” “rather often,” “very often or always.”

§§ categories were slightly different for the two items covering emotional demands: “to a very great extent, “to a great extent,” “to some extent,” “not really,” and “not at all”. Furthermore for item x, four categories was used.,

§§ Response categories “yes” or “no”. If “yes” – Can you estimate how much of the workday you do this? “almost all the time”, “about 3/4 of the time”, “about half the time”, “about 1/4 of the time”, “very little part of the time”
